# Expression and significance of SIRT6 in human peritoneal dialysis effluents and peritoneal mesothelial cells

**DOI:** 10.1007/s11255-024-03970-5

**Published:** 2024-03-14

**Authors:** Shuai-Shuai Shi, Yi-Qiang Zhang, Lu-Qi Zhang, Yun-Feng Li, Xiao-Shuang Zhou, Rong-Shan Li

**Affiliations:** 1 Department of Nephrology, Fifth Hospital of Shanxi Medical University (Shanxi Provincial People’s Hospital), Taiyuan, Shanxi China; 2 Department of Nephrology, Heji Hospital of Changzhi Medical College, Changzhi, 046011 Shanxi China; 3 Department of Biochemistry, Changzhi Medical College, Changzhi, 046000 Shanxi China; 4 First Clinical Department of Changzhi Medical College, Changzhi, 046000 Shanxi China

**Keywords:** Peritoneal dialysis, SIRT6, Mesothelial-to-mesenchymal transition, TGF-β1, Peritoneal fibrosis, Peritoneal mesothelial cells

## Abstract

Sirtuin 6 (SIRT6) can inhibit the fibrosis of many organs. However, the relationship between SIRT6 and peritoneal fibrosis (PF) in peritoneal dialysis (PD) remains unclear. We collected 110 PD patients with a duration of PD for more than 3 months and studied the influence of PD duration and history of peritonitis on SIRT6 levels in PD effluents (PDEs). We also analyzed the relationship between SIRT6 levels in PDEs and transforming growth factor beta 1 (TGF-β1), IL-6, PD duration, peritoneal function, PD ultrafiltration (UF), and glucose exposure. We extracted human peritoneal mesothelial cells (HPMCs) from PDEs and measured the protein and gene expression levels of SIRT6, E-cadherin, vimentin, and TGF-β1 in these cells. Based on the clinical results, we used human peritoneal mesothelial cells lines (HMrSV5) to observe the changes in SIRT6 levels and mesothelial-to-mesenchymal transition (MMT) after intervention with PD fluid. By overexpressing and knocking down SIRT6 expression, we investigated the effect of SIRT6 expression on E-cadherin, vimentin, and TGF-β1 expression to elucidate the role of SIRT6 in mesothelial-to-epithelial transition in PMCs. Results: (1) With the extension of PD duration, the influence of infection on SIRT6 levels in PDEs increased. Patients with the PD duration of more than 5 years and a history of peritonitis had the lowest SIRT6 levels. (2) SIRT6 levels in PDEs were negatively correlated with PD duration, total glucose exposure, TGF-β1, IL-6 levels, and the dialysate-to-plasma ratio of creatinine (Cr4hD/P), but positively correlated with UF. This indicates that SIRT6 has a protective effect on the peritoneum. (3) The short-term group (PD ≤ 1 year) had higher SIRT6 and E-cadherin gene and protein levels than the mid-term group (1 year < PD ≤ 5 years) and long-term group (PD > 5 years) in PMCs, while vimentin and TGF-β1 levels were lower in the mid-term group and long-term group. Patients with a history of peritonitis had lower SIRT6 and E-cadherin levels than those without such a history. (4) After 4.25% PD fluid intervention for HPMCs, longer intervention time resulted in lower SIRT6 levels. (5) Overexpressing SIRT6 can lead to increased E-cadherin expression and decreased vimentin and TGF-β1 expression in HPMCs. Knocking down SIRT6 expression resulted in decreased E-cadherin expression and increased vimentin and TGF-β1 expression in HPMCs. This indicates that SIRT6 expression can inhibit MMT in HPMCs, alleviate PF associated with PD, and have a protective effect on the peritoneum.

## Introduction

The incidence rate of end-stage renal disease (ESRD) is increasing year by year, which brings huge social and economic burden. Peritoneal dialysis (PD) is one of the renal replacement treatments for ESRD patients. Due to its low mortality rate and good preservation of residual kidney function, more and more patients are choosing peritoneal dialysis. However, some patients are unable to undergo long-term peritoneal dialysis, with the most serious reason being peritoneal dysfunction [1]. The short-term causes of peritoneal dysfunction are infections (mainly peritonitis) and catheter problems [2, 3], while in the long run, the main problem is peritoneal fibrosis (PF) caused by various reasons. Therefore, considering that peritoneal dialysis has higher economic and environmental sustainability than peritoneal dialysis, it is necessary to find new strategies to alleviate peritoneal fibrosis.

SIRT6 is a member of the silent information regulatory factor 2 family (sirtuins), mainly located in nuclear heterochromatin and plays an important role in DNA repair, genomic stability, metabolism, aging, oxidative stress, inflammation, and fibrosis [4]. It can inhibit fibrosis in many organs, such as the heart, kidneys, liver, and lungs [5]. However, its role in peritoneal fibrosis related to peritoneal dialysis is still unclear. Therefore, we investigated the relationship between SIRT6 and peritoneal function and peritoneal fibrosis.

## Materials and methods

### Patient selection

Patients with PD who were followed up regularly from July 2009 to May 2022 and underwent stable dialysis for more than 3 months in our PD center were included in the study. We excluded patients with tumors, infectious diseases, connective tissue disease, heart failure, abdominal bleeding, long-term use of immunosuppressive drugs, uncontrolled abdominal skin infection, combined hemodialysis treatment, patients with peritoneal dialysis-related peritonitis in recent 3 months, and discontinued PD. We collected and recorded the sex, age, basic disease, glucose exposure, peritoneal equilibration test (PET), and PD duration of all the included patients. The study was reviewed and approved by the Ethics Committee of Changzhi Medical College, and all patients provided informed consent.

### Diagnostic criteria for peritoneal dialysis-related peritonitis

2022 update on prevention and treatment Peritoneal Dialysis International [6]. Peritonitis should be diagnosed when at least two of the following are present: (1) Clinical features consistent with peritonitis, that is abdominal pain and/or cloudy dialysis effluent; (2) Dialysis effluent white cell count > 100/mL or > 0.1 × 10^9^/L (after a dwell time of at least 2 h), with > 50% polymorphonuclear leukocytes (PMN); (3) Positive dialysis effluent culture.

### Calculate the total glucose exposure

The calculation method for glucose exposure every 24 h is as follows: If a patient’s PD treatment plan consists of 3 bags of 2 L 1.5% low calcium peritoneal dialysis solution and 1 bag of 1.5 L 2.5% low calcium peritoneal dialysis solution, the exposure amount is: 3 × 2 × 15 + 1 × 1.5 × 25 = 127.5 (g), which means a glucose exposure of 127.5 g within 24 h. The total glucose exposure is obtained by multiplying the 24 h glucose exposure by the duration of peritoneal dialysis.

### PDEs’ samples

We collected PDEs from 2 L 2.5% glucose PD solution at 4 h and 4 weeks after recovery from PD patients, respectively. Dialysate samples were centrifuged at 3000 rpm for 15 min at 4 °C, aliquoted, and stored at − 80 °C.

### PET

The 2 L 2.5% glucose peritoneal dialysate was collected after 4 h, and the dialysate-to-plasma ratio of creatinine (Cr4hD/P) was measured. The ultrafiltration (UF) of the patient was measured 4 h after PD.

### ELISA assay

Samples were taken at the end of routine peritoneal equilibration tests (PET) to ensure a standardized method and well-mixed representative sample of dialysate. Dialysate cytokine levels were measured using enzyme-linked immunosorbent assay. The dialysate levels of SIRT6, TGF-β1, and IL-6 were measured using ELISA (Cat No: LV10933S, Aiming Youning, Shanghai, China). The ELISA results were analyzed using nonlinear regression with GraphPad Prism version 7.0 (GraphPad Software Inc., La Jolla, CA, USA).

### Primary cell culture and HmrSV5 cell line

PDEs were collected from patients undergoing PD therapy. Cells were pelleted from the PDEs using centrifugation at 1500 rpm for 10 min at 4 °C within 2 h after collection from the patients, followed by washing twice with phosphate buffer solution (PBS) The cells were cultured in Dulbecco’s modified Eagle medium/Ham’s nutrient mixture F12 (DMEM/F12) containing 10% fetal bovine serum (FBS) and 1% penicillin/streptomycin at 37 °C in a 5% CO_2_ incubator. Non-adherent cells were removed at each passage of the cell culture. Human peritoneal mesothelial cell line HmrSV5 was purchased from Chemical Book Company.

### Cell transfection

The small interfering RNA si-sirt6 and control were purchased from Ribo Biotechnology (Guangzhou, PR China). HmrSV5 cells were transfected with the si-sirt6 or control. The transfection complex was prepared by adding 1.25 μl of 20 μM si-sirt6 storage solution to 30 μl of riboFECTTMCP Buffer (cat # R10035.7 Ribobio), and this was followed by the addition of 3 μl of riboFECTTMCP Reagent and incubation for 5 min at room temperature. Transfection complexes were added to cells in complete medium and mixed, and the cells were used 48 h after transfection. pEGFP-N1-SIRT6 overexpression plasmid was transfected into HmrSV5 cells according to the instruction of manufacturer. The cells were used after 48 h transfection.

### Cell treatment with PDS or LPS

The HmrSV5 cells were treated with 4.25% peritoneal dialysis solution (PDS) for 24 h or 72 h. Then, the cells were used for qPCR and western blot analysis.

The HmrSV5 cells were treated with 1 ug/ml or 10 ug/ml LPS for 72 h. Then, the cells were used for qPCR and western blot analysis.

### Immunofluorescence analysis of cell surface markers

After three passages (P3), the expression levels of a panel of peritoneal mesothelial cell surface markers were measured using immunofluorescence with a specific antibody. Cells were seeded onto coverslips in 6-well plate for 24 h. The coverslips were washed thrice with PBS. Coverslips were fixed with 4% paraformaldehyde for 15 min. The cells were permeabilized in 0.5% Triton X-100 for 20 min, blocked with goat serum for 30 min, and incubated with a rabbit anti-vimentin polyclonal antibody (Cat No:10366-1-AP, Proteintech Group, Wuhan, China) overnight at 4 °C. Following three washes in PBS, the cells were incubated with Alexa Fluor-labeled secondary antibodies for 1 h at room temperature. Nuclei were stained with 4′, 6-diamidino-2-phenylindole.

### Scanning electron microscopy (SEM) of peritoneal mesothelial cells

Scanning electron microscopy (SEM) is an innovative method and was used to illustrate the morphological features of peritoneal mesothelial cells. Cells were fixed with freshly prepared 2.5% glutaraldehyde in DPBS at 4 °C for 3 h. Fixed cells were washed twice with DPBS and dehydrated with methanol. The stored dishes were brought to room temperature and washed once with DPBS.

Dehydration was carried out sequentially in the dishes with methanol at concentrations of 20, 40, and 60% for 5 min each, followed by an 80% methanol wash for 3 min, and a 100% methanol wash for 30 s, repeated five times. Coverslips with dishes were dried in vacuum-assisted desiccators overnight and stored at room temperature until SEM analysis was carried out. The surface of the coverslip was sputter-coated in vacuum with an electrically conductive 5 nm-thick layer of gold–palladium alloy precision etching coating system. Peritoneal mesothelial cell characteristics, such as margins, shapes, and surfaces, were observed and photographed.

### Real-time quantitative PCR technology for RNA extraction

Total RNA was extracted from PMCs or HmrSV5 cell using TRIzol reagent, and cDNA was synthesized using TB Green Rremix Ex Taq™ (cat. #RR820A, Takara). mRNA expression was measured on a fluorescence quantitative PCR according to RT-PCR kit instructions through the two-step amplification procedure: first step: one cycle at 95 ℃ for 30 s; second step: 40 cycles at 95 ℃ for 5 s and at 60 ℃ for 34 s. Primer sequences are shown in Table 1.

**Table 1:** Primers used to conduct mRNA expression analysis

Primer name	Primer sequence	Product size (bp)
β-actin	F:5′-CTCCATCCTGGCCTCGCTGT-3′	268
	R:5′-GCTGTCACCTTCACCGTTCC-3′	
E-cadherin	F:5′- CGGGAATGCAGTTGAGGATC-3′	201
	R:5′- AGGATGGTGTAAGCGATGGC-3′	
Vimentin	F:5′- TGCCAACCGGAACAATGAC-3′	71
	R:5′-ACTGCACCTGTCTCCGGTACTC-3′	
TGF-β1	F:5′- CGACTACTACGCCAAGGA -3′	150
	R:5′- GAGAGCAACACGGGTTCA -3′	
SIRT6	F:5′-CCCGGATCAACGGCTCTATC-3′	136
	R:5′-GCCTTCACCCTTTTGGGGG-3′	

### Western blot analysis

Peritoneal mesothelial cells or HmrSV5 cells were collected and the cells were lysed after tryptic digestion. The total cellular protein was extracted and subjected to BCA quantification. Thirty grams of protein was analyzed using SDS-PAGE, transferred onto membrane, blocked using 5% skim milk, bound with the primary antibody E-cadherin (1:2000), vimentin (1:2000), TGF-β1 (1:2000), SIRT-6 (1:2000), and α-tubulin (1:1000), and incubated at 4 °C overnight. Afterward, membrane was washed and incubated with goat anti-rabbit HRP-labeled secondary antibody (1:2000) for 2 h. Western blotting images were captured using Chemidoc XRS + (Bio-Rad) with enhanced chemiluminescence substrate and quantified using ImageJ.

### Statistical analysis

All statistical analyses were conducted using SPSS version 21.0. Normal distribution variables are represented as mean ± standard deviation (SD), while non-normal distribution variables are represented as median (interquartile spacing). Comparisons were conducted between single and two groups using *t* tests, and between multiple groups using one-way ANOVA and LSD tests. Multiple factors were analyzed using a multivariable model. The correlation between normal distribution variables (Pearson correlation), non-normal distribution variables, and rank variables (spearman correlation) was evaluated. The *P* value is set to *P* < 0.05.

## Results

### Patient characteristics

According to the inclusion and exclusion criteria, a total of 110 peritoneal dialysis patients were included, including 57 males and 53 females, with an average age of (53.64 ± 14.69) years. The duration of peritoneal dialysis was 7–125 months. According to the duration of peritoneal dialysis, it is divided into short-term group (PD ≤ 1 year), long-term group (PD > 5 years), and middle-term group (1 year < PD ≤ 5 years). According to whether the patient has a history of peritonitis, they are divided into a group with a history of peritonitis and a group without a history of peritonitis. Therefore, it is divided into six groups, group 1: short term and no history of peritonitis; group 2: middle term and no history of peritonitis; group 3: long term and no history of peritonitis; group 4: short term and history of peritonitis; group 5: middle term and history of peritonitis; group 6: long term and history of peritonitis. There is no statistically significant difference in gender, age, and incidence rate of diabetes in each group (Table 2).

**Table 2:** General situation of peritoneal dialysis patients

	No history of peritonitis			History of peritonitis			*P*
	Short term (group 1)	Middle term (group 2)	Long term (group 3)	Short term (group4)	Middle term (group 5)	Long term (group 6)	
Number	11	63	12	3	15	6	
Male (*n*, %)	5 (45.4)	31 (49.2)	8 (66.6)	1 (33.3)	8 (53.3)	3 (50)	0.876^b^
^a^Age (years)	50.6 ± 10.6	51.4 ± 6.1	59.9 ± 10.6	64.0 ± 4.6	54.3 ± 14.5	63.3 ± 7.3	0.14^c^
Glomerulonephritis	5 (45.3)	25 (39.7)	6 (50)	2 (66.7)	6 (40)	2 (33.3)	0.917^b^
Diabetic nephropathy	3 (27.3)	12 (19)	4 (33.4)	0	5 (33.4)	2 (33.3)	0.629^b^
Hypertension	2 (18.2)	10 (15.9)	1 (8.3)	0	2 (13.3)	1 (16.7)	0.952^b^
Polycystic kidney	1 (9.1)	6 (9.5)	0	1 (33.3)	0	0	0.293^b^
Others	1 (9.1)	10 (15.9)	1 (8.3)	0	2 (13.3)	1 (16.7)	0.938^b^
Dialysis method	Continuous abdominoperitoneal dialysis	CAPD	CAPD	CAPD	CAPD	CAPD	
Type of peritoneal dialysis solution	Changfu peritoneal dialysis solution (lactate-G1.5/2.5%)	Changfu peritoneal dialysis solution (lactate-G1.5/2.5%)	Changfu peritoneal dialysis solution (lactate-G1.5/2.5%)	Changfu peritoneal dialysis solution (lactate-G1.5/2.5%)	Changfu peritoneal dialysis solution (lactate-G1.5/2.5%)	Changfu peritoneal dialysis solution (lactate-G1.5/2.5%)	
^a^CRE (μmol/L)	812.90 ± 123.57	903.74 ± 184.42	894.08 ± 172.77	920.00 ± 51.96	853.20 ± 142.64	1076.16 ± 149.86	0.070^c^
^a^HB (g/L)	100.72 ± 11.67	100.49 ± 15.04	101.25 ± 13.67	105.33 ± 7.09	104.06 ± 11.42	99.83 ± 8.58	0.928^c^
^a^ALB (g/L)	33.81 ± 3.57	34.34 ± 4.14	35.33 ± 5.17	37.33 ± 2.08	34.73 ± 3.15	35.00 ± 2.09	0.768^c^
^a^CRP (mg/L)	4.12 ± 2.08	3.86 ± 1.95	4.01 ± 1.82	3.33 ± 0.65	3.66 ± 1.88	4.16 ± 1.90	0.977^c^

^a^Results presented as mean ± SD

^b^Inter-group comparison using Fisher’s exact test

^c^Inter-group analysis of variance test

### Using a single-factor multivariate model to analyze the impact of the duration of peritoneal dialysis and history of peritonitis on the level of sirt6 in PDEs

We used ELISA assay to detect the levels of SIRT6 in the effluent of 110 peritoneal dialysis patients. Compare groups based on different durations of peritoneal dialysis and whether there is a history of peritonitis. The duration of peritoneal dialysis has a significant impact on the main effect of sirt6 (*P* = 0.000). With the prolongation of peritoneal dialysis, sirt6 levels were significantly decreased. The presence or absence of a history of peritonitis had a significant impact on the main effect of sirt6 (*P* = 0.003). Peritonitis can cause a significant decrease in sirt6 levels.

**Fig. 1:**
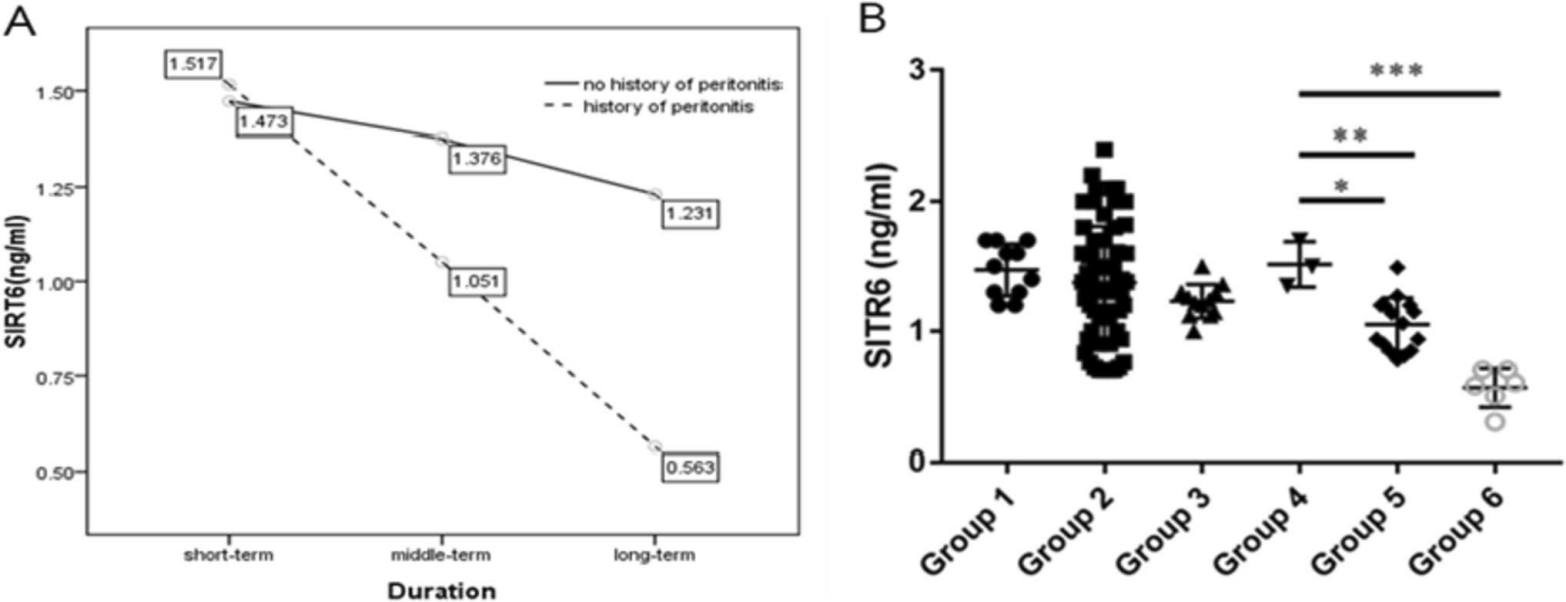
**A** The interaction between duration of peritoneal dialysis and history of infection on SIRT6 levels in PDEs. **B** Divided into six groups based on the duration of peritoneal dialysis and whether there is a history of infection, the levels of SIRT6 in PDEs in each group. *P < 0.05; **P < 0.01; ***P < 0.001 in comparison with the control group

**Fig. 2:**
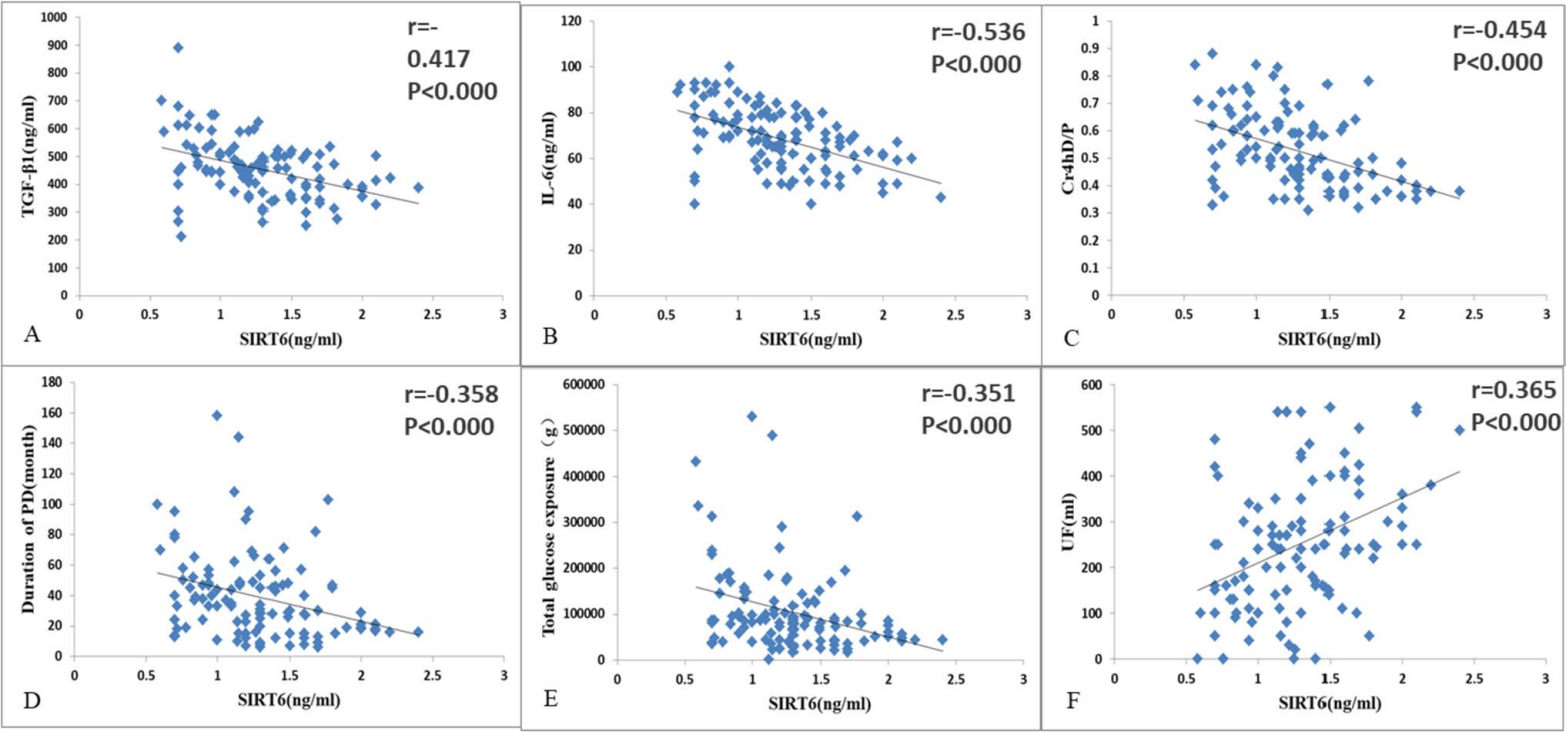
**A** Correlation analysis between SIRT6 level and TGF-β1. **B** Correlation analysis between SIRT6 level and IL-6. **C** Correlation analysis between SIRT6 level and Cr4hD/P. **D** Correlation analysis between SIRT6 level and peritoneal dialysis duration. **E** Correlation analysis of SIRT6 and total glucose exposure. **F** Correlation analysis of SIRT6 and UF

The interaction between peritoneal dialysis time and history of peritonitis on SIRT6 levels is statistically significant (*P* = 0.049). With the prolongation of peritoneal dialysis, the decrease in SIRT6 levels is more pronounced in patients with a history of peritonitis (Figs. 1 and 2).

### Correlation analysis between SIRT6 and IL-6 T, GF-β1, PD duration, total glucose exposure, Cr4hD/P, and UF

We used ELISA assay to detect the levels of IL-6 and TGF-β1 in the PDFs of 110 peritoneal dialysis patients, and collected clinical indicators from all patients, including PD duration, total glucose exposure, Cr4hD/P, and UF.

In all PD patients, SIRT6 levels in PDEs were negatively correlated with PD duration and total glucose exposure (Fig. 2D, E), and were negatively correlated with IL-6 and TGF-β1 expression in PDEs (Fig. 2A, B). Cr4hD/P was negatively correlated with SIRT6, whereas UF was positively correlated. (Fig. 2C, F).

### Identification of PMCs in PD patients

**Fig. 3:**
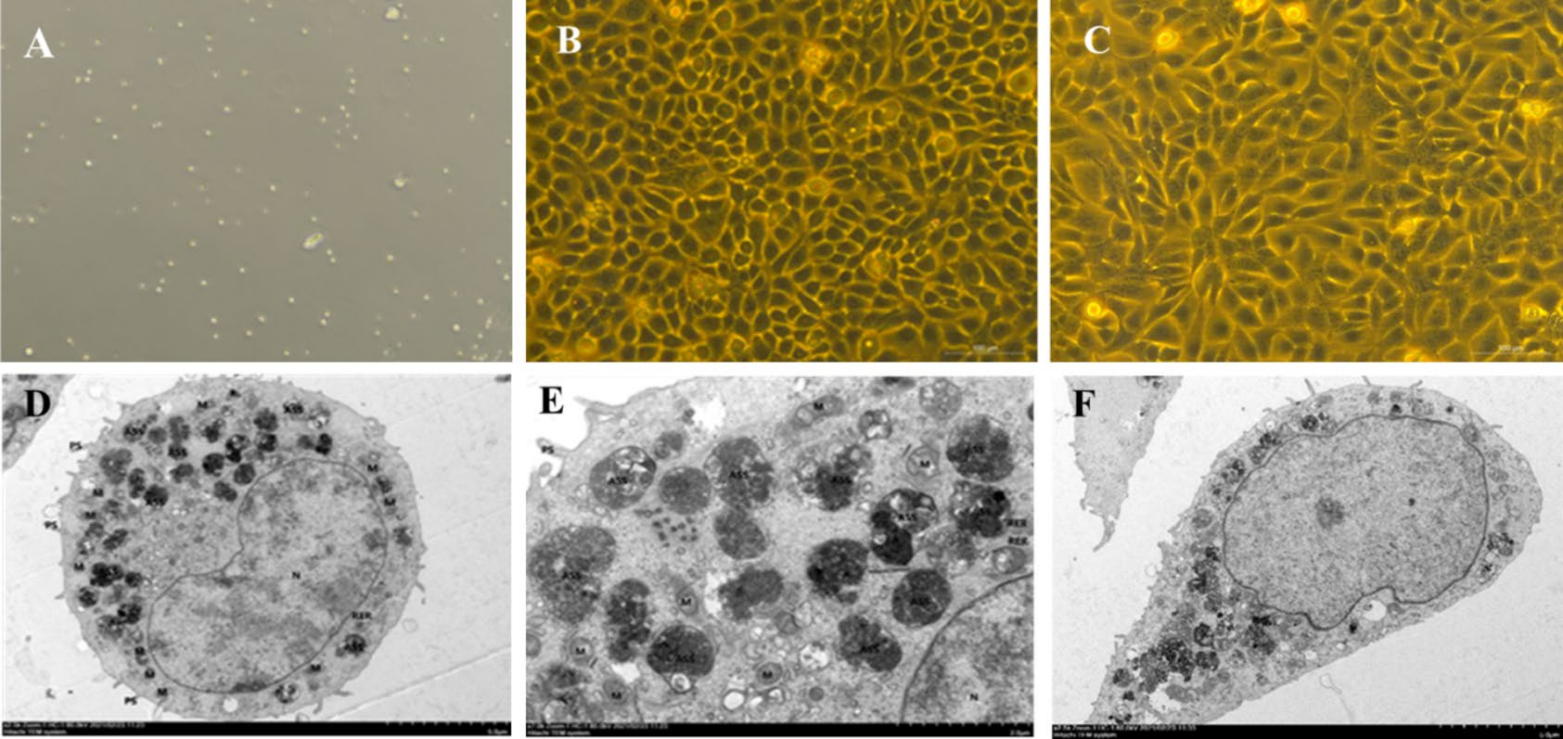
**A** Primary culture lens of PMCs 100 × . **B** PMCs in non-infected group were cultured for 14 days under light microscope × 100. **C** PMCs in infection group were cultured for 14 days under light microscope × 100. **D** Under transmission electron microscope, PMCs in group 1. **E** No Weibel–Palade body was found under transmission electron microscope. **F** Under transmission electron microscope, PMCs in group 6

Under light microscopy, most PMCs cultured by PDEs were epithelioid cells (Fig. 3A), The PMCs in the primary culture of PDEs in group 1 were cultured for 14 days, and the cells were fused into polygons, forming a paving stone-like appearance (Fig. 3B), whereas in group 6, the primary cultured PMCs showed a spindle shape (Fig. 3C).Under TEM, abundant filamentous microvilli were observed on the cell surface of the PMCs (Fig. 3D). Transmission electron microscopy showed abundant endoplasmic reticulum and mitochondria in the cytoplasm, but no Weibel–Palade bodies were found (Fig. 3E). The cells in the peritonitis infection group showed moderate edema, a long spindle shape, and intact cell membranes (Fig. 3F).

**Fig. 4:**
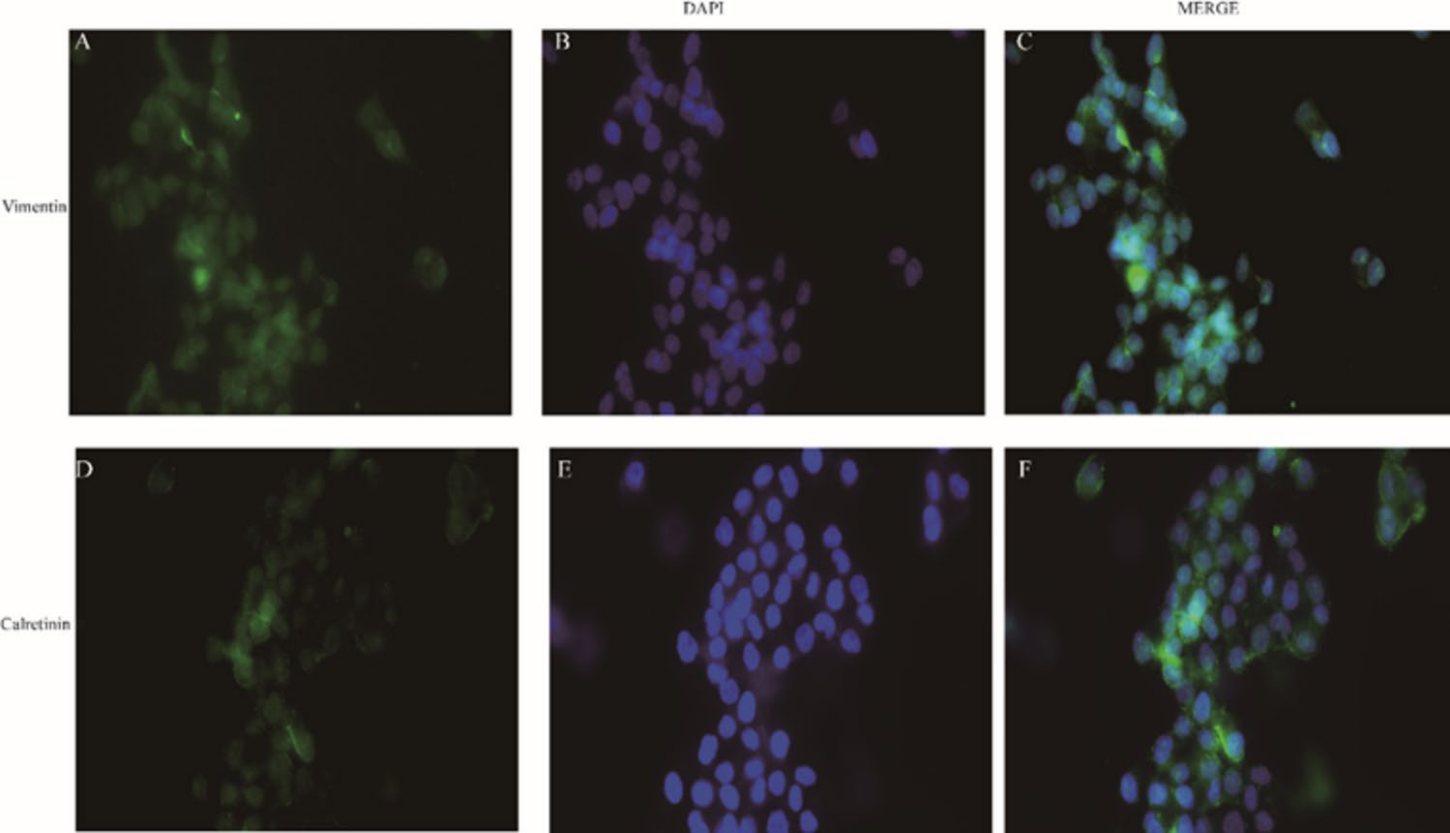
**A**–**C** HPMCs’ cell cytoplasm vimentin positive (× 200). **D**–**F** HPMCs’ cell cytoplasmic calretinin antigen positive (× 200)

Immunofluorescence showed that both vimentin and calretinin antigens were present in the cytoplasm and nucleus of PMCs (Fig. 4).

### Expression level of SIRT6, E-cadherin, vimentin, and TGF-β1 in PMCs

**Fig. 5:**
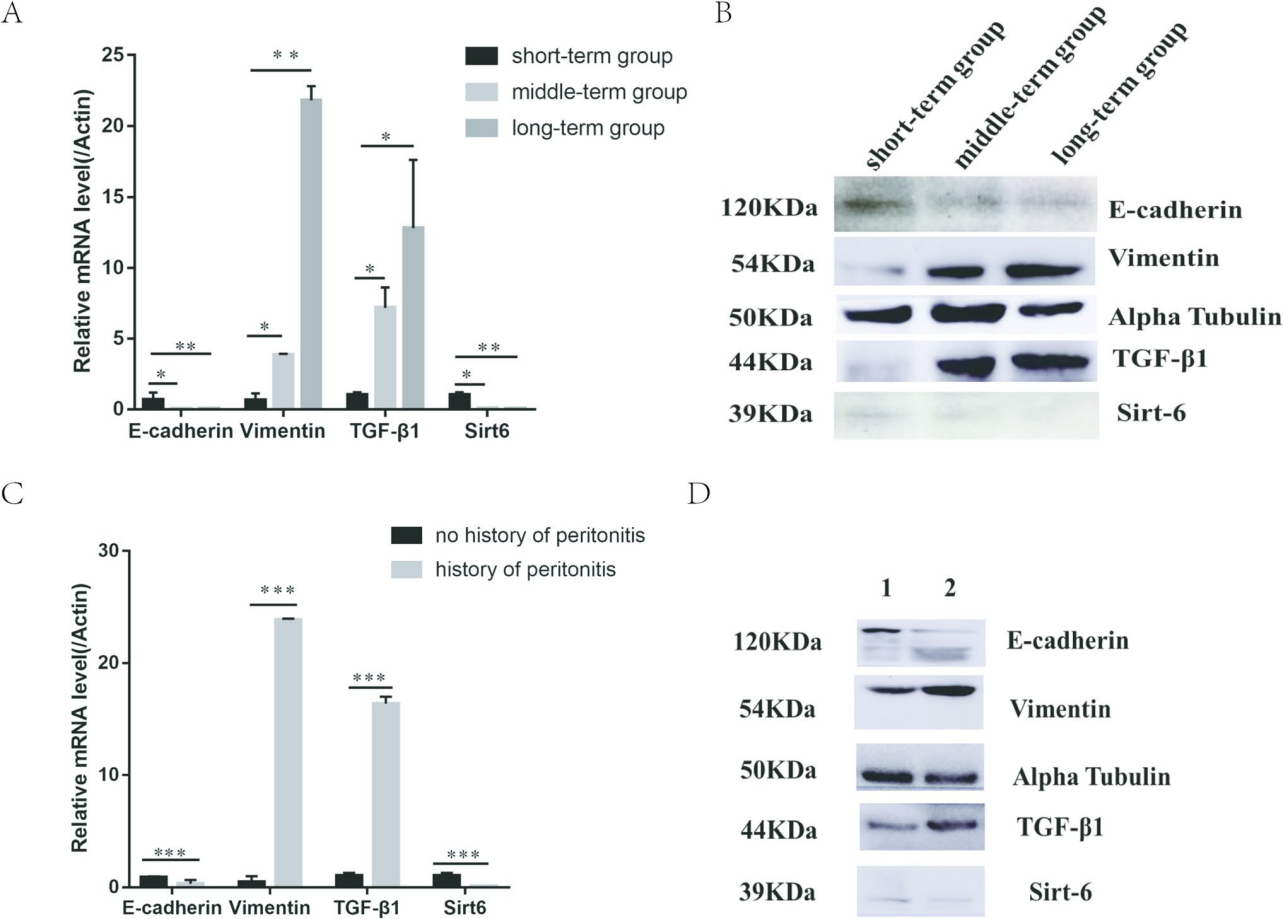
**A**. Gene levels of SIRT6, E-cadherin, vimentin, and TGF-β1 in PMCs of PD patients in short-term group, middle-term group, and long-term group. **B** The protein expression of SIRT6, E-cadherin, vimentin, and TGF-β1 in PMCs of PD patients in short-term group, middle-term group, and long-term group. **C** The gene levels of SIRT6, E-cadherin, vimentin, and TGF in PMCs in no history of peritonitis and history of peritonitis group. **D** The protein levels of SIRT6, E-cadherin, vimentin, and TGF-β1 of PMCs in no history of peritonitis and history of peritonitis group; *, *P* < 0.05; **, *P* < 0.01; and ***, *P* < 0.001 in comparison with the control group

In PD patients, the protein and gene expression levels of SIRT6 and E-cadherin in the short-term group were significantly higher than those in the long-term group and middle-term group, while the levels of vimentin and TGF-β1 were significantly lower than those in the long time group and middle time group, respectively (*P* < 0.05). However, the protein and gene expression levels of SIRT6, E-cadherin, vimentin, and TGF-β1 did not differ between the mid-term and long-term groups (*P* > 0.05) (Fig. 5A, B). Compared with the group without a history of peritonitis, the protein and gene levels of vimentin and TGF-β1 in the group with a history of peritonitis were significantly increased, while the protein and gene expression levels of SIRT6 and E-cadherin were significantly reduced (*P* < 0.05) (Fig. 5C, D).

### Cell treatment with PDS

**Fig. 6:**
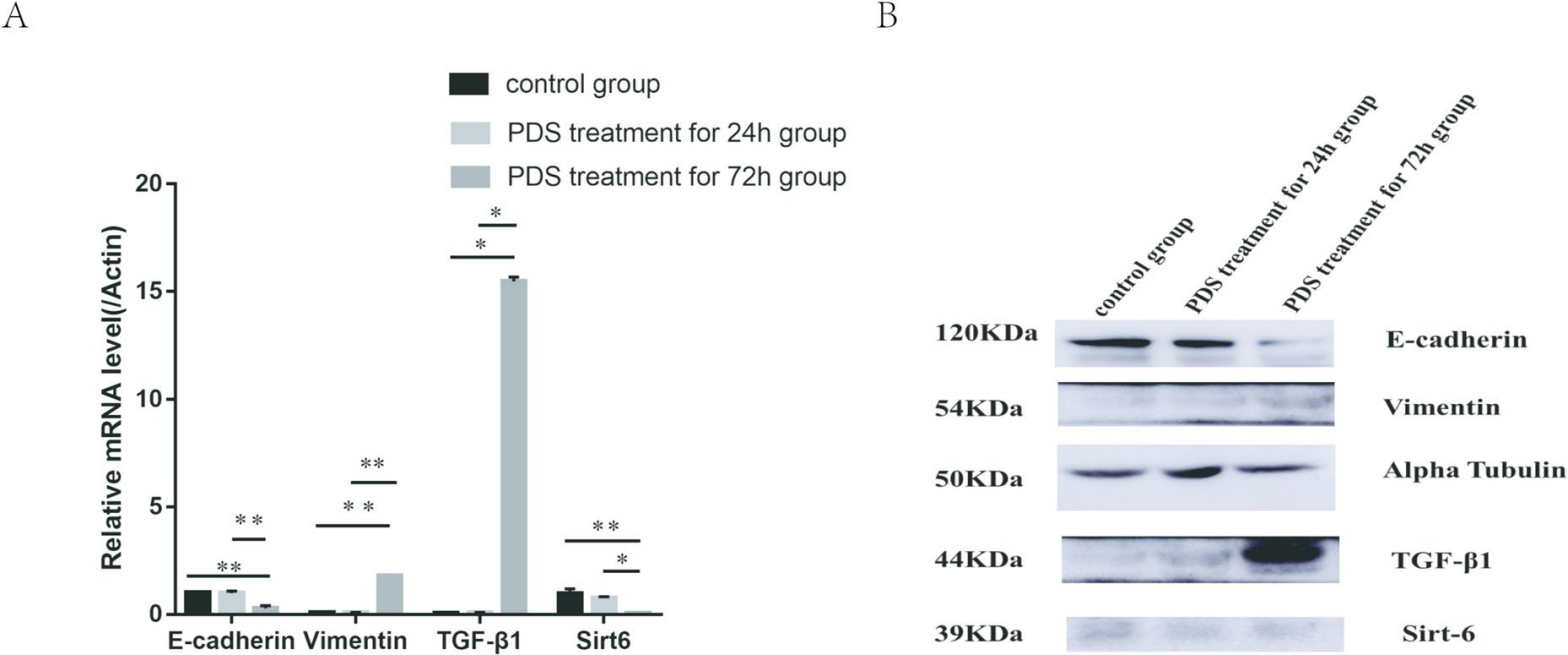
HMrSV5 cells were cultured with 4.25% PDS for 24 and 72 h. **A** The expression levels of SIRT6, E‑cadherin, vimentin, and TGF-β1 were detected using western blot analysis. **B** The gene levels of SIRT6, E‑cadherin, vimentin, and TGF-β1. *P < 0.05; **P < 0.01; and ***P < 0.001 in comparison with the control group

HMrSV5 cells were treated at different times with 4.25% peritoneal dialysis solution (PDS). The results showed that there was no difference in the levels of SIRT6, E-cadherin, vimentin, and TGF-β1 between the 24 h PDS treatment group and the control group. The levels of SIRT6, E-cadherin, vimentin, and TGF-β1 in the 72 h PDS treatment group showed significant statistical differences compared to the 24 h PDS treatment group and control group. The gene and protein levels of SIRT6 and E-cadherin in the 72 h PDS treatment group were significantly reduced, while the gene and protein levels of vimentin and TGF-β1 were significantly increased (Fig. 6).

### Cell treatment with LPS

**Fig. 7:**
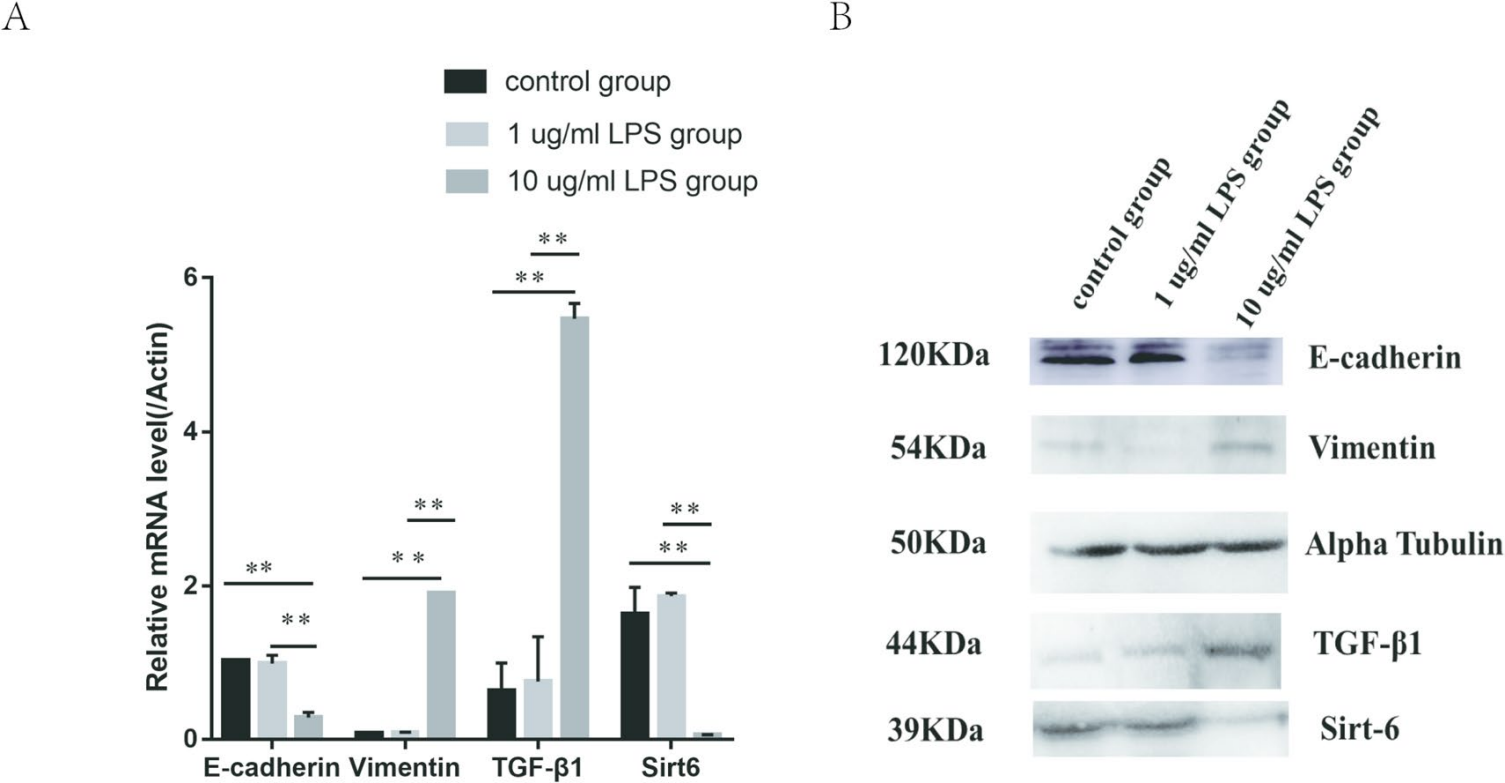
Intervention HMrSV5 cells with 1ug/ml and 10ug/ml LPS. **A** The expression levels of SIRT6, E‑cadherin, vimentin and TGF-β1 were detected using western blot analysis. **B** The gene levels of SIRT6, E‑cadherin, vimentin and, TGF-β1. *P < 0.05; **P < 0.01; and ***P < 0.001 in comparison with the control group

HMrSV5 cells were treatment with 1 ug/ml or 10 ug/ml LPS. The results showed that there was no difference in the levels of SIRT6, E-cadherin, vimentin, and TGF-β1 between the 1 ug/ml LPS group and the control group. The levels of SIRT6, E-cadherin, vimentin, and TGF-β1 in the 10 ug/ml LPS group showed significant statistical differences compared to the 1 ug/ml LPS group and control group. The gene and protein levels of SIRT6 and E-cadherin in the 10 ug/ml LPS group were significantly reduced, while the gene and protein levels of vimentin and TGF-β1 were significantly increased (Fig. 7).

### The effect of overexpression or knockdown of SIT6 in HMrSV5 cells.

**Fig. 8:**
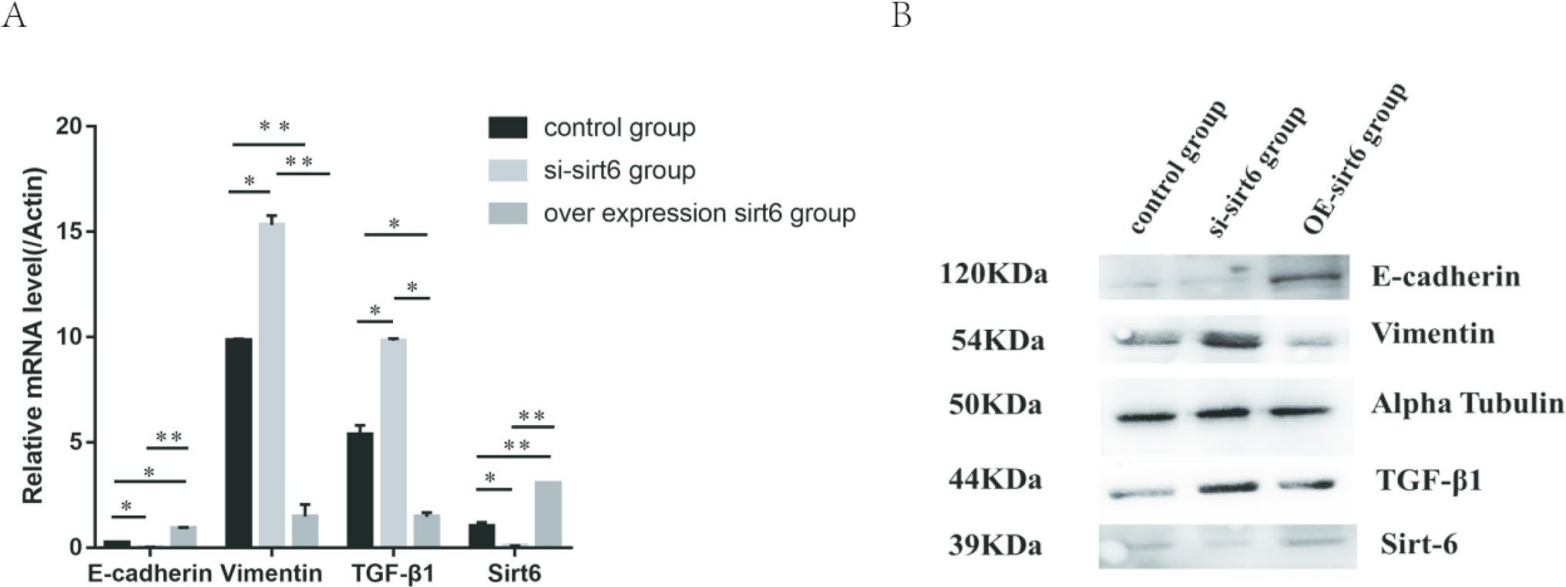
After transfection with plasmids and small interfering RNA, human peritoneal mesothelial cells were treated with 4.25% peritoneal dialysis solution to investigate the protein and gene levels of SIRT6, E-cadherin, vimentin, and TGF- β1 in the cells. **A** The gene levels of SIRT6, E-cadherin, vimentin, and TGF-β1 in HPMCs. **B** The protein levels of SIRT6, E-cadherin, vimentin, and TGF-β1 in HPMCs. *P < 0.05; **P < 0.01; and ***P < 0.001 in comparison with the control group

Preprocess HMrSV5 cells with pEGFP-N1-SIRT6 plasmid and small interfering RNA, overexpress or knock down SIRT6 expression, and intervene again with 4.25% peritoneal dialysis solution to observe the changes in SIRT6, E-cadherin, vimentin, and TGF-β1.The results showed that there were statistically significant differences in the expression of SIRT6, E-cadherin, vimentin, and TGF-β1 among the overexpression group, knockdown group, and control group. The overexpression of SIRT6 group had the highest levels of E-cadherin and the lowest levels of vimentin and TGF-β1. The knockdown SIRT6 group had the lowest levels of E-cadherin and the highest levels of vimentin and TGF-β1 **(**Fig. 8**).**

## Discussion

The main reason why peritoneal dialysis patients cannot undergo long-term peritoneal dialysis is due to various reasons leading to peritoneal dysfunction. It is urgent to find treatment methods to improve peritoneal dysfunction. SIRT6 is a member of the silent information regulator 2 family (sirtuins), located on chromosome 19p13.3 and containing 355 amino acid residues with a molecular weight of approximately 39.1 kDa12 [7]. It is mainly located in nuclear heterochromatin and is related to many diseases, such as cancer, inflammatory diseases, diabetes, steatohepatitis, arthritis, cardiovascular diseases, neurodegenerative diseases, virus infection, and corneal injury [8]. There is no research on whether SIRT6 is related to peritoneal dialysis-related peritoneal dysfunction.

We collected clinical data from 110 patients at our peritoneal dialysis center, including Cr4hD/P and peritoneal dialysis ultrafiltration volume, and studied the relationship between SIRT6 and peritoneal function. Correlation analysis confirmed that the level of SIRT6 is negatively correlated with Cr4hD/P and positively correlated with peritoneal dialysis ultrafiltration volume. At the same time as the decrease in peritoneal clearance of creatinine and ultrafiltration capacity, the level of SIRT6 is also decreasing. It indicates that SIRT6 is closely related to peritoneal function and may have a protective effect on peritoneal function.

Research suggests that the causes of peritoneal dysfunction include the following: biocompatibility of peritoneal dialysis fluid, formation of glycation end products, uremic toxins, dialysis-related peritonitis, and chronic inflammation induced by inflammatory mediators [9]. The non-physiological characteristics and uremic state of PD fluid are considered the main factors leading to decreased peritoneal function. These factors can trigger chronic peritonitis, and the onset of peritonitis can exacerbate this inflammation [10].

Based on the common causes of damage to peritoneal function, we grouped patients into different durations of peritoneal dialysis and whether they had a history of peritonitis. We applied a multivariate analysis method to analyze the impact of peritoneal dialysis duration and history of peritonitis on SIRT6. Research has found that patients with a history of peritonitis and peritoneal dialysis lasting for more than 5 years have the most significant decrease in SIRT6 levels in PDEs. Correlation analysis also found that the level of SIRT6 is negatively correlated with peritoneal dialysis time and total peritoneal glucose exposure. Patients with a history of peritonitis had significantly lower levels of SIRT6 compared to patients without a history of peritonitis. The reasons that can lead to peritoneal dysfunction also lead to a decrease in SIRT6 levels, further confirming that the decrease in SIRT6 expression occurs in the same direction as the decrease in peritoneal function.

The most important pathological change leading to peritoneal dysfunction is peritoneal fibrosis. At present, it has been found that the mechanisms leading to peritoneal fibrosis include MMT in peritoneal mesothelial cells, increased extracellular matrix production, angiogenesis, and transforming growth factors β1 (TGF-β1) Overexpression, induction of renin angiotensin activation, and increased expression of fibronectin (FN) [11–13]. SIRT6 can inhibit the occurrence and development of fibrosis in various organs, including the heart, liver, lungs, kidneys, etc. The mechanisms of anti-fibrosis include: Recombinant Mothers Against Decapitaleptic Homolog 2 (Smad2) direct interaction, which affects the acetylation level and transcriptional activity of Smad2 [14]. Regulating the expression of fibrosis promoting target genes downstream of TGF-β1/Smad signaling pathway [15]. Negatively regulate the activity of cyclin dependent kinase (CDK) and E2F transcription factors, inhibiting the occurrence and development of fibrosis [16]. Inhibition nuclear factor κ B (NF-κ B), thereby inhibiting inflammatory responses and fibrosis processes [17]. Inhibiting the production of reactive oxygen species (ROS) in cells and protecting cells from oxidative stress damage [18]. Regulating the occurrence and development of fibrosis by regulating autophagy [19]. At the same time, fibrosis can also be inhibited by inhibiting the occurrence of EMT [20]. It is not yet known whether SIRT6 can affect peritoneal fibrosis and thus affect peritoneal function.

TGF-β1signaling is a common mediator of peritoneal fibrosis [21]. IL-6 is also considered an important cytokine in peritoneal fibrosis, leading to peritonitis and fibrosis development through the STAT3-dependent pathway [22]. We first investigated the relationship between SIRT6 levels in PDFs of peritoneal dialysis patients and TGF-β1, IL-6. Correlation analysis found a negative correlation between SIRT6 levels and levels of TG-β1F and IL-6. The decrease in SIRT6 levels may be involved in the process of peritoneal fibrosis.

Among the numerous mechanisms of peritoneal dialysis-related peritoneal fibrosis, the MMT of PMCs is a special type of EMT, which is the initial and reversible link of peritoneal fibrosis. Therefore, the MMT of peritoneal mesothelial cells is currently a hot research topic, and it is believed that EMT can serve as an early intervention target for peritoneal integrity [23]. Multiple signaling molecules can activate MMT, and epigenetic changes, microRNAs, and post translational modifications can also regulate the occurrence of MMT.

Similarly, research has shown that SIRT6 can affect the progression of fibrosis by affecting EMT. Huang et al. [8] found that SIRT6 whole gene knockout mice can develop chronic nephritis and progress to renal fibrosis at 7 months of age. Research has found that overexpression of SIRT6 can significantly inhibit the epithelial–mesenchymal transformation of TGF-β1-induced human lung cancer cell line A549, while overexpression of SIRT6 can negatively regulate the transformation of TGF-β1-induced human fetal lung fibroblasts (HFL1) into myofibroblasts [24]. Sangeeta Maity et al. [5] found that SIRT6 deficiency plays a major role in the aging related transformation of fibroblasts into myofibroblasts, leading to tissue fibrosis. There are also research results, indicating that SIRT6-deficient fibroblasts spontaneously transform into myofibroblasts through over activation of TGF-β1 signals in a cellular autonomous manner. For example, in the liver, lungs, myocardium, and other tissues, the knocking out of SIRT6 in specific organs can lead to increased fibrosis, and in lung and liver tissues, Inhibition of TGF-β1/Smad3 and NF-k B/Snail signaling pathways [25]. Therefore, we speculate that SIRT6 inhibits peritoneal fibrosis by inhibiting MMT in PMCs.

To confirm this hypothesis, we collected peritoneal mesothelial cells from peritoneal dialysis effluent and detected the protein and gene expression of SIRT6, E-cadherin, vimentin, and TGF-β1 in the cells. The experiment found that compared with other dialysis patients, peritoneal dialysis patients with a history of peritonitis had significantly lower levels of SIRT6 and E-cadherin in peritoneal mesothelial cells compared to patients without a history of peritonitis, while levels of vimentin and TGF-β1 were higher than those without a history of peritonitis. Grouping based on the duration of peritoneal dialysis, it was found that the levels of SIRT6 and E-cadherin in the short-term group were significantly higher than those in long-term dialysis patients, while the levels of vimentin and TGF-β1 were lower than those in long-term dialysis patients. The trend of changes in levels of SIRT6, E-cadherin, vimentin, and TGF-β1 indicates that prolonged dialysis time and a history of peritonitis lead to a decrease in SIRT6 and changes in MMT in peritoneal mesothelial cells. Meanwhile, we designed a cell experiment using 4.25% peritoneal dialysis solution and LPS to intervene in HMrSV5 cells. As the intervention time of peritoneal dialysis fluid prolonged, the levels of SIRT6 and E-cadherin were gradually decreased, while the levels of vimentin and TGF-β1 were gradually increased. High concentrations of LPS can lead to EMT in HMrSV5 cells, while the level of SIRT6 significantly decreases. This is consistent with the clinical trial results. This indicates a negative correlation between changes in SIRT6 levels and changes in MMT, confirming the correlation between SIRT6 and MMT in peritoneal mesothelial cells, which may be an inhibitory factor for MMT changes.

To further verify the relationship between SIRT6 and MMT in peritoneal mesothelial cells, we used plasmids and small interfering RNA to pretreat HMrSV5 cells to overexpress or decrease SIRT6 expression. Then, 4.25% peritoneal dialysis solution was used to intervene in HMrSV5 cells to investigate the relationship between SIRT6 and MMT in human peritoneal mesothelial cells. Experiments have shown that HMrSV5 cells overexpressed with SIRT6, under the intervention of peritoneal dialysis fluid, have significantly reduced MMT status, manifested by an increase in E-cadherin levels and a decrease in vimentin levels. However, in HMrSV5 cells with reduced SIRT6 expression, under the intervention of 4.25% peritoneal dialysis solution, the MMT status was significantly worsened, with a more significant decrease in E-cadherin levels and a more severe increase in vimentin levels. The results confirmed that the expression of SIRT6 can inhibit MMT in human peritoneal mesothelial cells.

While demonstrating the relationship between SIRT6 and MMT, we investigated the relationship between SIRT6 and TGF-β1. Research has found that overexpression of SIRT6 can inhibit TGF-β1 expression, while a decrease in SIRT6 expression stimulates TGF-β1 expression. We speculate that SIRT6 may reduce the occurrence of MMT in human peritoneal mesothelial cells by downregulating the expression of TGF-β1.

Overall, our experiments have demonstrated a positive correlation between SIRT6 expression and peritoneal function in PD patients, while showing a negative correlation with MMT in PF and PMCs. These data provide evidence for the association between SIRT6 and peritoneal function and PF. The role of SIRT6 in PD-associated peritoneal fibrosis warrants further investigation. In our next step, we plan to further study the cell signaling transduction of SIRT6 in MMT of HPMCS, and verify the relationship between SIRT6 and peritoneal fibrosis and MMT in an animal model of peritoneal fibrosis. It may have the potential to resist peritoneal fibrosis and preserve peritoneal function, serving as a therapeutic target for improving peritoneal fibrosis.

## Data Availability

No data was used for the research described in the article.
